# Investigation of physicochemical and sensory characteristics of low calorie sponge cake made from flaxseed mucilage and flaxseed flour

**DOI:** 10.1038/s41598-023-47589-5

**Published:** 2023-11-28

**Authors:** Fariba Ahmadinia, Forogh Mohtarami, Mohsen Esmaiili, Sajad Pirsa

**Affiliations:** Department of Food Science and Technology, Faculty of Agriculture, Urmia University, Urmia, Iran

**Keywords:** Biochemistry, Health care, Chemistry

## Abstract

This research aimed to extract flaxseed mucilage (FM) and investigate its rheological properties (static and dynamic tests) compared with animal oil. In the next stage, the D-optimal design was applied to investigate the effect of FM (0–60%) and FF (0–30%) replacements with animal oil and cake flour, respectively on the sponge cake's physicochemical, textural, and sensory properties. According to the flow behavior test, FM showed Newtonian behavior while animal oil had pseudoplastic behavior. The results of the dynamic test showed with an increase in frequency, the loss modulus (G״) and storage modulus (Gʹ) of samples increased. However, Gʹ was higher than G״ in all samples. By replacement of FM and FF, the moisture content, water activity, antioxidant capacity, crumb hardness, and cohesiveness of the samples increased while springiness, crust hardness, and specific volume decreased (P < 0.05). Lightness of samples with replacement of FF decreased (P < 0.05). The overall acceptance score was enhanced with an increase in FM substitution while it was decreased with the replacement of FF (P < 0.05). The amounts of fat, calories, and peroxide values were decreased in sponge cake with the incorporation of FF and FM (P < 0.05). In general, the substitution of FM (60%) and FF (28%) in the cake formulation as an optimized sample to make new products with low-calorie content is possible without significant decreases in product quality.

## Introduction

The bakery industry is expanding rapidly, and its products are gaining popularity among all segments of society. Cakes, in particular, are a highly sought-after snack due to their wider consumer base, convenience, and delicious taste. As a caloric source, the sponge cake is in the middle of bread and biscuits. Vegetable and animal oils can be used in the sponge cake formula. Also, fats are the main factor in improving the taste of sponge cakes^1,2^. Getting a lot of fat causes an increase in all types of cancer, and getting saturated fat causes an increase in blood cholesterol and coronary artery disease. Also, consumption of food rich in fat has been identified as a risk factor for receiving more energy and spreading obesity. To achieve better health, it is recommended to increase the consumption of fruits, vegetables, and legumes and modify the type and amount of fat consumed^3^. Therefore, with the increase in people's demand to reduce fat in the diet, food product manufacturers started producing new substitutes to replace most of the fats in foods. Fat replacers not only provide some or all of the functional properties of fat, but also have fewer calories than the fat being replaced, and are used in a wide range of products from baked goods to frozen desserts^4^. Fat substitutes can be based on protein and carbohydrates^5,6^. Mucilage, gums, starch, pectin, cellulose, and other carbohydrate substances provide many functions of fat in food by bonding with water and creating a favorable texture and mouthfeel^4,7–9^. The most important substitutes for fat in bakery products are mucilage and gum. Flaxseed is one of the most important functional foods containing high amounts of alpha-linolenic acid, lignan, dietary fiber, protein, minerals, flavonoids, phenolic acids, and vitamins^4,10^. It contains 24 to 44% oil, of which 50 to 55% is alpha-linolenic acid. Alpha-linolenic acid as a rich source of omega-3 (ω-3) is an essential fatty acid, especially for people who do not consume fish^11,12^. Linoleic acid constitutes approximately 16% of the total fatty acids present in flaxseed. The ratio of ω-3 to ω-6 in flaxseed oil varies from 1 to 0.3. Meanwhile, in most edible oils, the amount of ω-6 is more than ω-3. This distribution of fatty acids in flaxseed oil is nutritionally valuable^13^. The outer layers of flaxseed contain a remarkable amount of mucus or gum, setting it apart from other oil seeds and cereals^4^.

Therefore, due to the consumers' awareness of health problems, there is a significant demand for low-fat, low-calorie, and enriched foods. In this regard, many kinds of research have been conducted on the use of fat mimics such as psyllium mucilage and flaxseed mucilage^4^, modified rice starch^14^ in cookies and pumpkin powder^15^, and pureed butter beans^16^ in sponge cake, psyllium^17^ in gluten-free cookies, inulin, and resistant dextrin^18^ in gluten-free biscuit, chia seed mucilage^19^ in bread and sponge cakes. Enrichment of products with various flours has been also investigated by many researchers^20–28^.

Considering the things mentioned above, the use of FM and FF in food production can increase the nutritional value of the resulting products. In the literature review, no study was also found regarding the formulation of low-calorie sponge cakes using FM and FF. Therefore, this study aimed to investigate the rheological properties of extracted FM with different ratios of flaxseed and water were examined and compared to animal oil used in sponge cake formulation. Subsequently, the selected FM was incorporated as a fat substitute with different levels of FF in sponge cake production, and their sensory, physicochemical, and texture properties were evaluated.

## Materials and methods

### Chemicals and devices

Flaxseeds were purchased from the herbal medicine pharmacy in Urmia (Iran). 2,2-diphenyl-1-picrylhydrazyl (DPPH), methanol, hydrochloric acid, petroleum ether, sulfuric acid, copper sulfate, selenium dioxide, boric acid, sodium sulfate, chloroform, and other required compounds to tests were purchased from Merck (Germany) and Sigma-Aldrich (USA). Animal oil (prepared from animal butter) with 80% fat, cake flour, eggs, powdered sugar, vanilla, and nonfat dry milk were purchased from the local market in Urmia (Iran).

### Preparation of flaxseed mucilage and flaxseed flour

In a preliminary study for preparing FM as an oil substitute, different ratios of flaxseed with water were prepared, and a ratio having the same consistency as semi-solid animal oil was to be used as an oil substitute in the sponge cake formulation.

**Figure 1:**
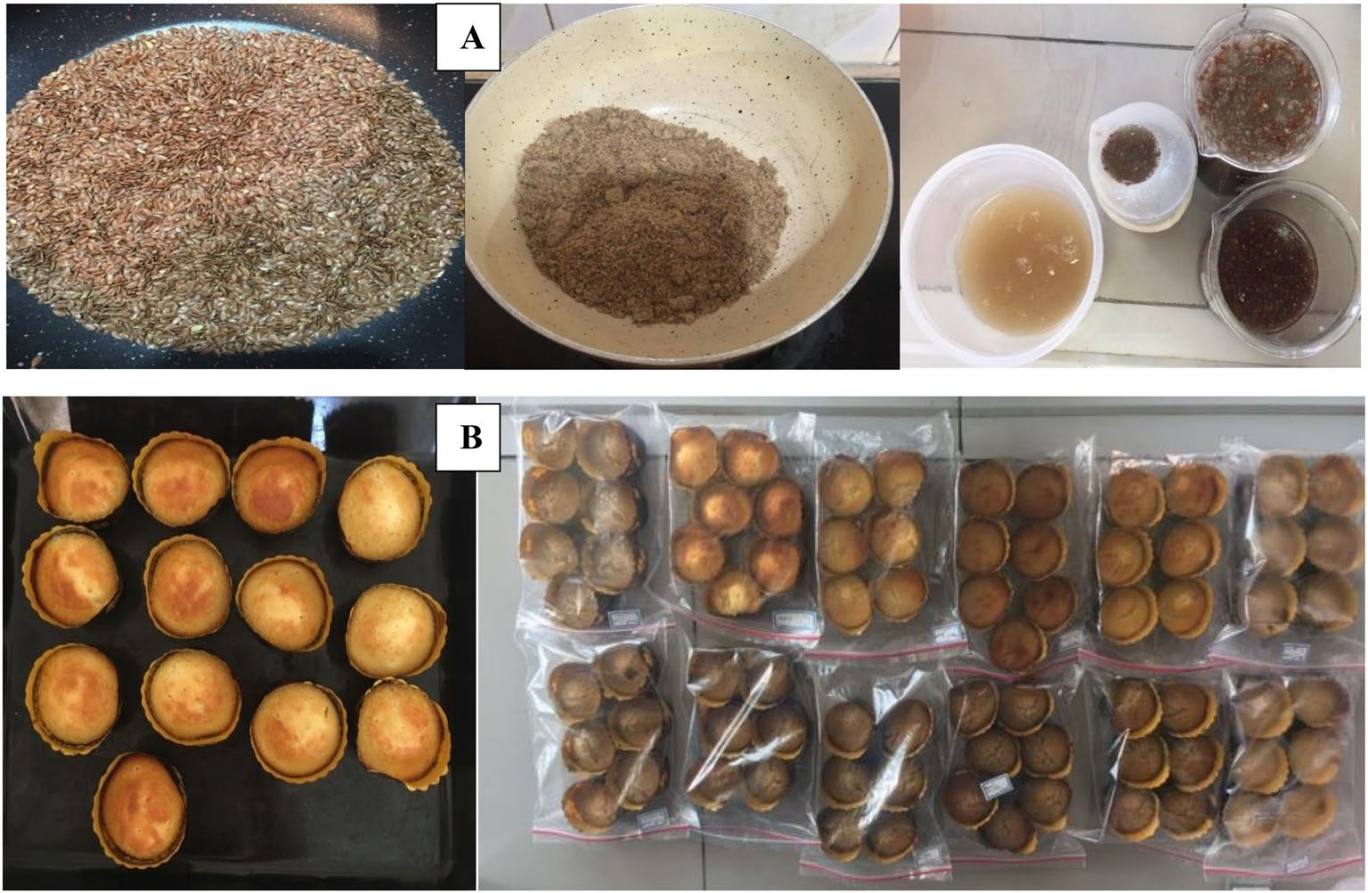
Flaxseed, flaxseed powder and mucilage (**A**) and low-calorie sponge cake sample (**B**).

In order to prepare mucilage, different proportions of flaxseed and water (1;40, 1:30, 1:25, 1:20, 1:15, and 1:10) were mixed and stirred at 50 °C for 2 h (Fig. 1A). The resulting mixture was passed through a mesh cloth in two steps to separate the insoluble components of the seed from the mucilage part^4^.

To prepare flaxseed powder, after cleaning, the flax seeds were roasted on a gentle heat and after cooling, they were powdered by a mill. The resulting powder was sieved to separate the coarse shell components^15^ (Fig. 1A).

### Preparation of sponge cake

The control sponge cake was prepared according to Mohtarami 2018^21^ with little modification. The formulation in this study was based on 100g wheat flour containing 60g sugar, 56g whole egg, 49 g animal oil, 3g nonfat dry milk, 3g baking powder, 1g vanilla, and 105g water. For this purpose, the whole egg and sugar were mixed at a higher disk speed of the mixer (KM 3067, Germany) for 1.5 min. Afterward, the water and animal oil were added and mixed for 1 min at middle speed followed by other ingredients. Then, 50 ± 1 g of obtained batter was poured into a paper mold (50 mm diameter × 40 mm height) and baked for 20 min at 200 °C in a conventional oven. After cooling for 1h at ambient temperature, sponge cakes were packed and stored in a polyethylene bag before analysis. According to the D-optimal design (Table 1), the FM (mucilage to water ratio, 1:20) and FF, as independent factors, were substituted with animal oil (0–60%) and sponge cake flour (0–30%) in other sponge cake samples, respectively (Fig. 1B).

**Table 1:** D-optimal design for substitution percent of Flaxseed mucilage with animal oil and Flaxseed flour with cake flour.

Run	A: Flaxseed flour (%)	B: Flaxseed mucilage (%)
1	15	15
2	0	60
3	30	0
4	0	0
5	30	30
6	30	60
7	15	60
8	0	60
9	0	30
10	30	60
11	7.5	45
12	0	0
13	30	0

### Determination of rheological characteristics

Rheological tests were carried out by MCR301 rheometer made by Anton Paar, Austria, using two parallel plates at a temperature of 20 °C. These tests included a static rheological test (flow behavior test) and a dynamic test (frequency sweep test).

#### Flow behavior test

This test was done to plot flow charts and identify the changes in apparent viscosity. Also, to detect the time-dependent behavior, the shear rate ranged from 0–250 s^−1^. To obtain an appropriate mathematical model for predicting the flow behavior of samples, shear stress versus shear rate data were fitted with the power law (1) and the Herschel-Bulkley model (2) as follows:

1 $$ \tau \,{ = }\,{\text{k}}\gamma^{{\text{n}}} $$

2 $$ \sigma \, = \,\sigma_{{\text{H}}} \, + \,{\text{K}}_{{\text{H}}} {\gamma }^{{^{{\text{n}}} }} $$

In this regard, τ is the shear stress (Pa), γ is the shear rate (s^−1^), K is the consistency coefficient (Pa.s^n^), σ_H_ is the yield stress and n is the flow behavior index (dimensionless). The consistency coefficient, the viscosity of the fluid, and the flow behavior index show how close the fluid behavior is to the Newtonian fluid^29^.

### Dynamic test

Dynamic tests were performed in the viscoelastic linear region and the loss modulus (G´´) and storage modulus (G´) were plotted as a function of shear rate and frequency. The frequency sweep test was performed in the frequency range of 0.1–100 Hz and by applying a constant strain of 1%. The curves obtained from the frequency sweep test were drawn according to the frequency (Hz). The three important parameters that result from this test are complex viscosity, storage modulus (G´), and loss modulus (G״). The storage modulus shows the amount of elastic behavior and the amount of energy stored in a volume unit and the loss modulus (G״) indicates the amount of flow behavior and the amount of dissipated energy per volume unit. In the frequency sweep test, if (G״) < (G´), the sample has solid viscoelastic behavior, and if (G״) > (G´), the sample shows liquid viscoelastic behavior^15^.

### Chemical tests

#### Moisture measurement

The moisture content of biscuits was determined using an air oven (Jeto Tech, OF-O2G) at the temperature of 130 °C for 1 h^30^. The moisture was calculated as follows (3):

3 $$\mathrm{Moistur} \left(\%\right)=\frac{M1-M2}{M0 }\times 100$$

M_1_ and M_2_ are the weight of the container and sample before and after drying and M_0_ is the weight of the sample.

#### Ash measurement

To determine the amount of ash, accurately weighed samples were incinerated in a porcelain crucible in a muffle furnace (FM4P) at 600 °C for approximately 3 h until forming a light ash^16^. The ash content was calculated according to the following formula (4):

4 $$Ash \left(\%\right)=\frac{M1-M2}{M0}\times 100$$

M_1_ is ash containing crucible weight and M_2_ is empty crucible weight and M0 is the weight of the sample.

#### Antioxidant activity

DPPH free radical scavenging method was used to determine the antioxidant activity of sponge cake samples. At first, 1 g of sponge cake sample was mixed with 10 ml of methanol and kept at room temperature away from light for 24 h. Then the supernatant was separated from the settled part and centrifuged for 10 min at high speed. 1 ml of the clear upper part (sample extract) was mixed with 4 ml of 90% methanol and 1 ml of DPPH methanol solution (0.004%) and placed in a dark place for half an hour. To determine the absorbance of the solution, the spectrophotometer was first calibrated at a wavelength of 517 nm with methanol, and then the absorbance of the samples was measured at the same wavelength^4^. Antioxidant capacity was calculated by the following equation (5):

5 $$Antioxidant \,acitivity \left(\%\right)=\frac{Ac-As}{Ac}\times 100$$

where A_S_ is the absorption rate of the sample and A_C_ is the absorption rate of the control.

#### Measurement of peroxide number

To measure the peroxide of the sponge cake samples, first, 2 g of the fat of the sponge cake samples was extracted and poured into a 250 ml flask. Then 30 ml of acetic acid and chloroform solution (2:3 volume ratio of acetic acid to chloroform) was added. Then, 0.5 ml of potassium iodide saturated solution was included in the solution and kept in a dark place for 1 min. Afterward, 30 ml of distilled water was added to the solution. At this stage, the obtained solution has a yellow color, and the titration should be continued until it becomes colorless. For this purpose, a few drops of starch solution were added to the solution. The prepared sample, which had a dark color, was titrated with 0.01 sodium thiosulfate until it became colorless. The amount of peroxide was calculated according to the following equation (6):

6 $$P \left(meq\frac{{O}_{2}}{kg}\right)=\frac{\mathrm{N }\times \mathrm{V }\times 100}{\mathrm{W}},$$

where N is sodium thiosulfate normality, V is the volume of sodium thiosulfate for titration (ml), and W is the weight of fat.

#### Protein measurement

Protein was measured using the Kjeldahl method (model 19,105) according to the method of AACC (2000), by considering nitrogen to a protein conversion factor of 6.25^31^. The used equation for calculation is presented as follows (7):

7 $$Nitrogen \left(\%\right)=\frac{N \times 0.014 \times \left({V}_{2}- {V}_{1}\right)}{m},$$

where N is the normality of consumed chloride acid, V_2_ is ml of acid used in the sample, V_1_ ml of acid used in the control, and m is the weight of the sample (g).

#### Fat measurement

The fat content was done by using the AACC-Approved method^31^ using the Soxhlet model P-5c in which some quantity of absolutely uniform and dried samples become wrapped in filter-out paper and located on a paper finger. Fat percentage was calculated from the following equation (8):

8 $$Fat (\%)\frac{ ({W}_{2}- {W}_{1})}{m}\times 100$$

where W_1_ and W_2_ are the initial weight of the empty balloon and the balloon contained fat, respectively and m is the weight of the sample.

#### Carbohydrate

Carbohydrate content was obtained by deducting the total moisture, fat, ash, and protein content of the samples from 100^20^.

#### Calorie evaluation

For this purpose, the percentage of fat, protein, and carbohydrate was multiplied by their energy-generating values, and the total calories of the samples were calculated according to the following equation (9):

9 $$ {\text{Calories }}\left( {\text{kcal/g}} \right) \, = \, \left( {{\text{fat}} \times 9} \right) + \left( {{\text{protein}} \times 4} \right) + \left( {{\text{carbohydrate}} \times 4} \right) $$

### Physical tests

The volume of cakes was determined by the rapeseed replacement method^32^ and then a specific volume was calculated. The water activity (aw) was determined using an MS1 water activity meter (Novasina, Lahen, Switzerland) after calibration with standard relative humidity salts. The temperature was maintained at 25 °C during the measurement. For calculating the baking loss weight, the weight of the dough was divided into baked sample weights^20^. To measure the weight loss of sponge cake dough after baking, the coded samples of sponge cake dough were weighed before and after baking, and then the weight loss was calculated according to the difference in the weight of the samples before and after baking^17^ which was determined by the following equation (10):

10 $$Weight \,loss (\%)\frac{ (Cake \,dough \,weight-Cake\, weight \,after \,baking)}{Cake\, dough \,weight}\times 100$$

### Color properties

The colors of the film samples were determined by the colorimeter (Minolta model CR-410, Tokyo, Japan). Results were expressed as lightness-darkness (L*), greenness-redness (a*), and blueness-yellowness (b*)^21^.

### Sensory evaluation

To evaluate the sensory characteristics of the sponge cake samples, the 5-point hedonic method was used to test the sponge cake samples on the first day of baking by 25 trained panelists in terms of color, taste, texture, smell, and overall acceptance of the item. In this test, the level of panelists' satisfaction with the sensory characteristics of sponge cake samples was collected and evaluated (1 = very poor, 2 = poor, 3 = moderate, 4 = good, 5 = very good)^33^.

### Texture analysis

The textural characteristics of the cakes were measured using a texture analyzer (TA-XT plus, Stable Micro System Ltd, Surrey, UK). For this purpose, samples of the central part of the cake (2 × 2 × 2 cm) were subjected to two consecutive compression tests as TPA (texture profile analyzer) using a 75 mm flat aluminum probe. Selected settings included a test speed of 0.8 mm/s with 50% compression and a 5 s time interval between the two cycles. The obtained texture parameters include crumb hardness (maximum force required for compression of the sample in the first cycle, Kg), cohesiveness (ratio of the area of the time force curve during the second compression to the first compression), springiness (time ratio of the second compression to the first compression), and chewiness (firmness × cohesiveness × springiness, kg). The crust firmness of the prepared sponge cake samples was done the first and seventh days after the baking. For this purpose, a 2 mm probe was used with a test speed of 1 mm/s. The maximum force of penetration was reported as crust firmness^21^.

### Statistical analysis

The effects of partial replacement of FF (0–30%) with cake flour and FM (0–60%) with animal oil were planned in 13 treatments by the response surface design (RMS) based on the D-optimal method (Table 1) in the formulation of sponge cake. The single effect and the interaction of these two factors were evaluated at the α = 5% level on the characteristics of the sponge cake. Design Expert-10 software was used to draw contour plots and obtain mathematical models. If models fitted to the experimental data were significant (p < 0.05) and had high R^2^ and adj-R^2^, and insignificant lack of fit, they could be used as more reliable models for predicting the effect of factors on the physical and sensory properties of the samples.

A numerical optimization function was performed to find the composition giving maximum desirability for chosen properties simultaneously i.e. maximum values for cohesiveness and overall acceptability and minimum values for crumb and crust hardness.

## Results and discussion

### Rheological properties

In the preliminary study for the preparation of FM as an animal oil substitute, different ratios of flaxseed to water (1:10, 1:15, 1:20, 1:25, 1:30, and 1:40) were prepared, and a ratio having a similar consistency to semi-solid animal oil was used as an animal oil substitute in the cake formulation. For this purpose, according to laboratory studies and visuals, it was found that the ratios of 1:10 of FM to water were much thinner than the animal oil (reached ambient temperature) and the ratios upper than 1:20 of FM were also much thicker than semi-solid animal oil. Therefore, ratios of 1:15 and 1:20 of FM to water were selected to determine rheological properties.

**Figure 2:**
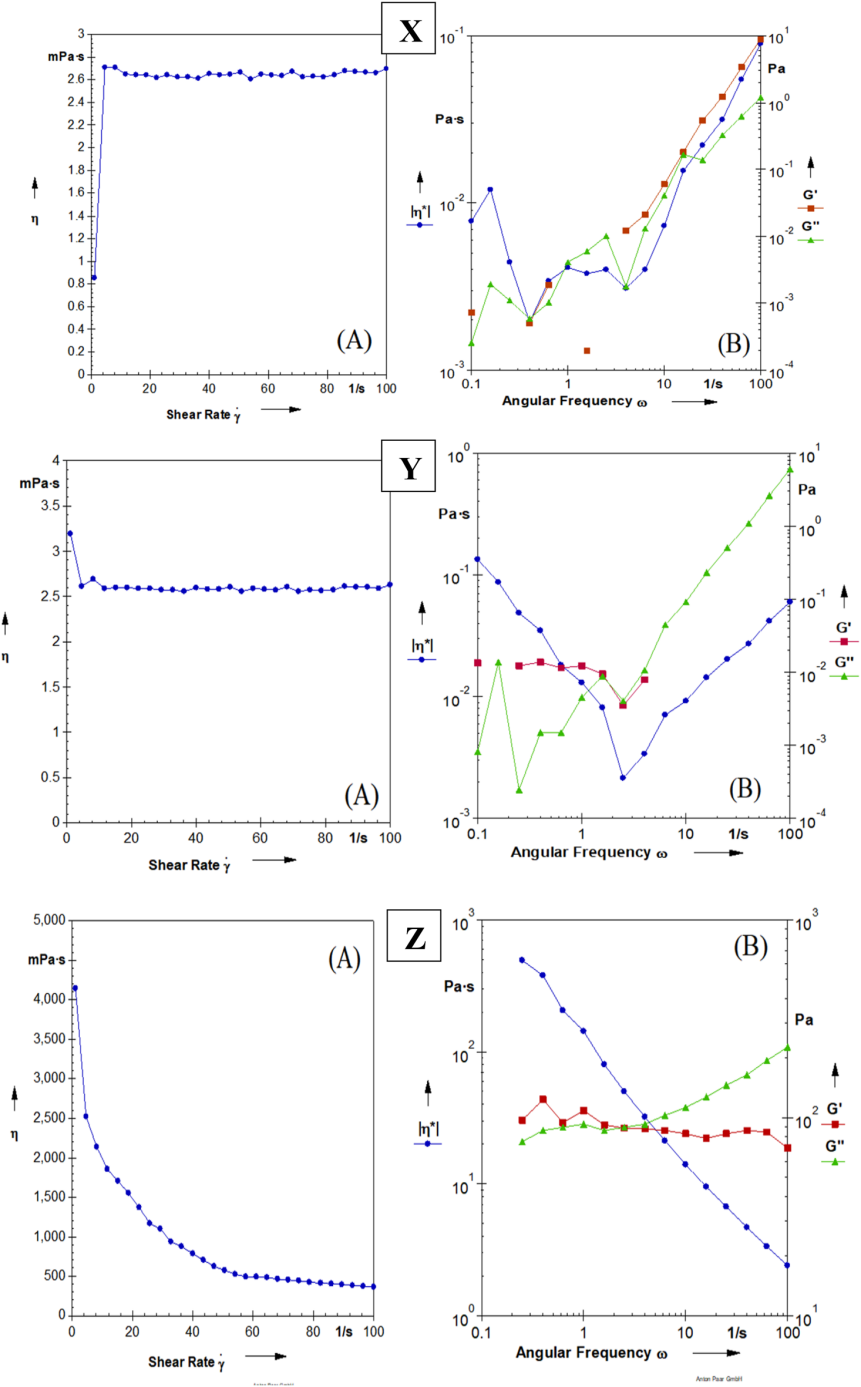
Viscosity diagram (**A**), frequency sweep of G´, G´´ and *ŋ (**B**) in the flax seed mucilage (1 to 15 ratio to water) (X), Flax seed mucilage sample (1:20 ratio to water) (Y) and animal oil sample (Z).

Figure 2 shows the apparent viscosity of FM (ratios 1:15 and 1:20 water) and animal oil. Apparent viscosity was almost constant with increasing shear rate in FM and animal oil samples. Constant viscosity with increasing shear rate indicates the Newtonian behavior in the samples. In the animal oil sample, the viscosity decreased with the increase in shear rate, but overall it had a higher viscosity than the FM samples. The decrease in viscosity with the increase in shear rate indicates the pseudoplastic behavior of animal oil. The reduction of apparent viscosity in animal oil has a higher intensity at the beginning of the increase in shear speed, but then the intensity of the diminish in apparent viscosity decreased and reached a constant value. The reason for the sharp decrease in apparent viscosity is due to the loss of intermolecular bonds. This is because the link between the molecules is broken and as a result, the apparent viscosity decreases slowly or becomes stable^34^. Similar results regarding Newtonian behavior have been reported about the rheological behavior of a variety of compounds such as corn fiber gum, modified corn starch, gum Arabic, and soluble soybean polysaccharides^35^. Based on the results, the apparent viscosity of modified starch and corn fiber gum solutions was almost constant due to highly branched structures and was independent of the shear rate, and no obvious pseudoplastic behavior was observed^35^. Cuomo et al. (2020) also investigated the behavior of chia seed mucilage combined with lemon essential oil and stated that the use of essential oil leads to the stability of the Newtonian behavior of chia mucilage^36^.

The frequency sweep test is the most common dynamic test. In this test, the amplitude of the input voltage or strain is kept constant, while the frequency is increased. The frequency sweep test is used to investigate the effect of different additives (for example, hydrocolloids) and the effect of different processes (for example, stirring and heating) on changing the viscoelastic behavior of materials. In this test, if the storage modulus (G') is larger than the loss modulus (Gʹʹ), it indicates a gel-like structure, and if the Gʹʹ is larger, the material in this area shows liquid characteristics. According to Fig. 2, with increasing frequency (0.1–1 Hz) at a constant strain of 1%, the Gʹʹ of all samples increased. In the FM with a ratio of 1:15 to water, the G' and Gʹʹ are increasing, which indicates the thin and semi-thinned behavior of solutions. There is no big difference between both modules, and both are frequency-dependent. Also, the distance between both modules has been maintained during different frequencies. In the FM sample with a ratio of 1:20, first, the G' is greater than the Gʹʹ, and then from a certain frequency onwards, the G' decreases and the Gʹʹ increases. In the FM sample with a ratio of 1:20, the Gʹ values are in a larger range than the G״ of the mucilage sample with 1:15, which is due to the increase in the concentration of the sample and as a result, the increase in the interaction of polymers, which causes an increase in the consistency of the sample. In the animal oil sample, the Gʹ is almost constant and has little dependence on the frequency, and the Gʹʹ increased significantly from a certain frequency onwards. So at higher frequencies, the distance between Gʹ and Gʹʹ increases, and the value of Gʹʹ is greater than Gʹ, which shows that the fluid-like behavior is increasing. In terms of units, the values ​​of Gʹ and Gʹʹ in the animal oil sample are between 10 and 1000 Pa, and in mucilage samples between 10^–4^ to 10 Pa. While the Gʹʹ and Gʹ values ​​in the animal oil sample are higher than in the mucilage samples. Another parameter investigated in the frequency test is the complex modulus, which is obtained from the ratio of the maximum stress to the maximum strain in the oscillation test. The complex modulus shows the overall stiffness, which includes elastic stiffness and viscous stiffness. The complex viscosity (µ^*^) is obtained from the ratio of the complex modulus to the frequency, and it is a measure of the overall stiffness of the desired material^37^. The µ^*^ in the mucilage samples first decreased and then increased; while in the animal oil sample, the µ^*^ decreased. However, µ^*^ was the greatest in the animal oil sample followed by FM (1:20 ratio) and FM (1:15 ratio), respectively. Based on the diagrams, FM with a ratio of (1:20) to water was stronger than FM with a (1:15) ratio to water and maintained its structure more during the tests. Therefore, it was chosen as a replacement for animal oil in the formulation of sponge cake. Table 2 presents the results of experimental data fitted with rheological models, namely the power law and Herschel-Bulkley. Based on the results, the power law model showed the best fit with the experimental data, as evidenced by the high determination coefficient. The coefficient of consistency (k) indicates the viscosity of the solution. FM (1:20) has a higher k value than FM (1:15) due to its higher molecular weight and greater hydrodynamic volume, which results in the formation of intermolecular bonds^4^. The animal oil sample displays a higher consistency coefficient than the mucilage samples, indicating a higher molecular weight. The flow behavior index (n) for the mucilage samples (1:15 and 1:20) is close to one, implying a Newtonian behavior while the flow behavior index for animal oil is less than one, which indicates pseudoplastic and non-Newtonian behavior in animal oil.

**Table 2:** Fitted models of power-law and Herschel-Bulkley with flaxseed mucilage (1:20 ratio to water) and animal oil.

Model	Animal oil	FM (1:20)	FM (1:15)
Power law model (σ = K_p_ ${\gamma }^{np})$			
K_p_	11.85	0.029	0.025
n_p_	0.24	0.99	1.00
R^2^	0.82	0.99	0.99
Hershel Bulkly model (σ = σH + K_H_ ${\gamma }^{np})$			
σ	− 31.24	74.89	73.08
K_H_	31.27	− 14.96	− 13.16
n_H_	0.02	− 0.01	− 0.01
R^2^	0.84	0.73	0.75

*K* consistency index, *n* flow behavior index, *R*^*2*^ determination coefficient.

### Moisture content and water activity (aw)

**Figure 3:**
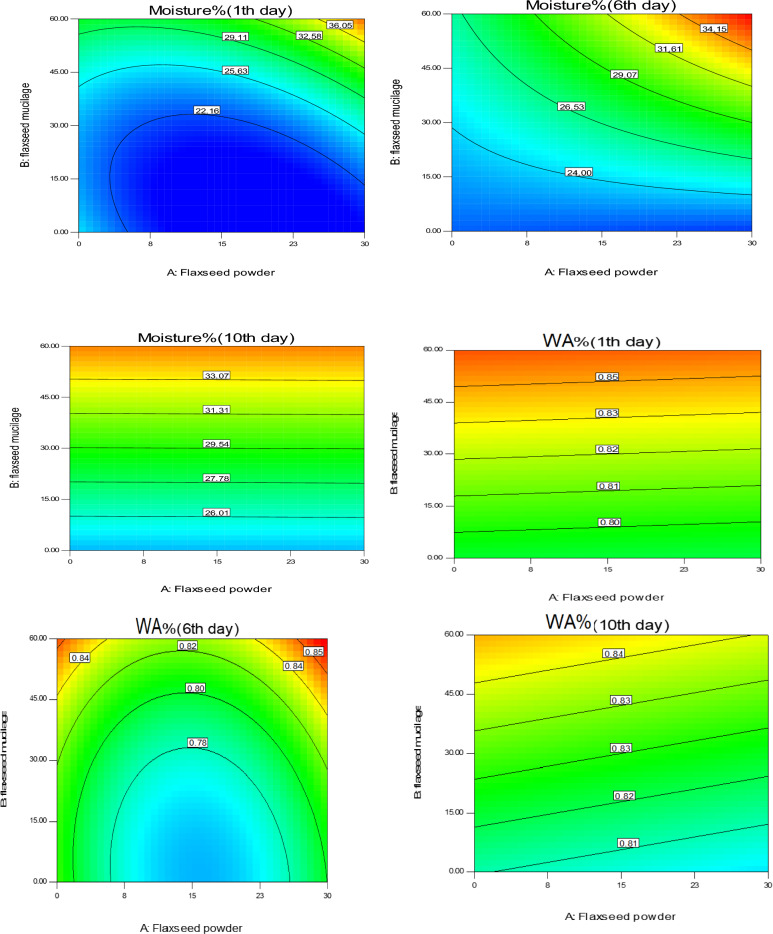
Contour plot of the effect of replacing flaxseed mucilage and flaxseed flour on the moisture content and water activity (WA) of the low-calorie sponge cake on the 1st, 6th, and 10th day after baking and the antioxidant capacity of the sponge cake.

Figure 3 shows the contour plot of the effect of replacing FM and FF on the moisture content and aw^38^ of the low-calorie sponge cake on days 1, 6, and 10 after baking. As shown in Fig. 3, with the increase of FM substitution, the moisture content of the samples increased. FF had no significant effect on moisture at low FM replacement, but at high levels of FM, with the increase of FF, the moisture content of the samples increased significantly. On the tenth day after baking, FF had no significant effect on moisture content, but with the increase of FM, the moisture content increased significantly (P ≤ 0.05). Mucilage can form a continuous gel-like texture, so they can successfully maintain moisture content during the shelf life. Compounds that can crosslink and create a gel, forming a three-dimensional network that traps water, can increase product moisture. On the other hand, the oil in food can prevent evaporation by surrounding water molecules, especially during the cooking process, and act as a barrier to the exit of moisture. The influence of FF in keeping water is more than that of oil, which increased the moisture content of sponge cake samples by reducing the amount of oil in the formulation. It is worth noting that the moisture content of sponge cakes containing FF increases more than those containing wheat flour. This is because FF contains a higher amount of minerals (1.54%) and crude fiber (1.80%) compared to wheat flour (0.62% minerals and 1.1% crude fiber). The increased amount of fiber in FF causes it to absorb more water, which in turn increases the moisture content of the cake^38^. Similar results have been reported with the addition of FM^4^, different kinds of hydrocolloids and gums^39,40^ in cookies, and chia seed mucilage in cake and bread^19^ as fat replacers. On the other hand, an increase of moisture content by incorporation of FF has been confirmed in gluten-free biscuits^20^ and cookies^41^.

The results of aw evaluation on the 1st, 6th, and 10th day after baking showed that with the replacement of FM, the aw increased significantly (P < 0.05), but FF had no significant effect on this parameter. In general, replacing animal oil with FM increased aw, which is consistent with the results of increasing moisture. The use of mucilage in food due to its hydrophilic properties leads to the creation of water-soluble molecules and controls the moisture content of the food through the formation of a structure that is permeable to water. The results of this research were in agreement with the results of 0ther studies^4,19,39^.

The predictive regression models for moisture and aw were a significant model with high R^2^ and Adj-R^2^, and a non-significant lack of fit (P > 0.05), which indicates the effectiveness of the predicated model. The presented models for moisture and aw on 1st, 6th, and 10th after baking are as follows (11–16):

11 $${\text{Moisture}} \left(1\text{th\, day}\right)=21.61+1.10\times A+6.13\times B+3.43\times AB+3.66\times {A}^{2}+3.57\times {B}^{2}$$

12 $${\text{Moisture}} \left(6\text{th \,day}\right)=26.58+2.48\times A+4.84\times B+34.96\times C+2.76\times AB$$

13 $${\text{Moisture}} \left(10\text{th \,day}\right)=29.54+0.03\times A+5.25\times B$$

14 $$\text{aw }\left(1\text{st \,day}\right)=0.82-0.001\times A+0.03\times B$$

15 $${\text{aw}} \left(6\text{th\, day}\right)=0.77+0.001\times A+0.02\times B+0.005\times AB+0.04\times {A}^{2}+0.01\times {B}^{2}$$

16 $${\text{aw}} \left(10\text{th\, day}\right)=0.8-0.003\times A+0.01\times B$$

### Antioxidant capacity

**Figure 4:**
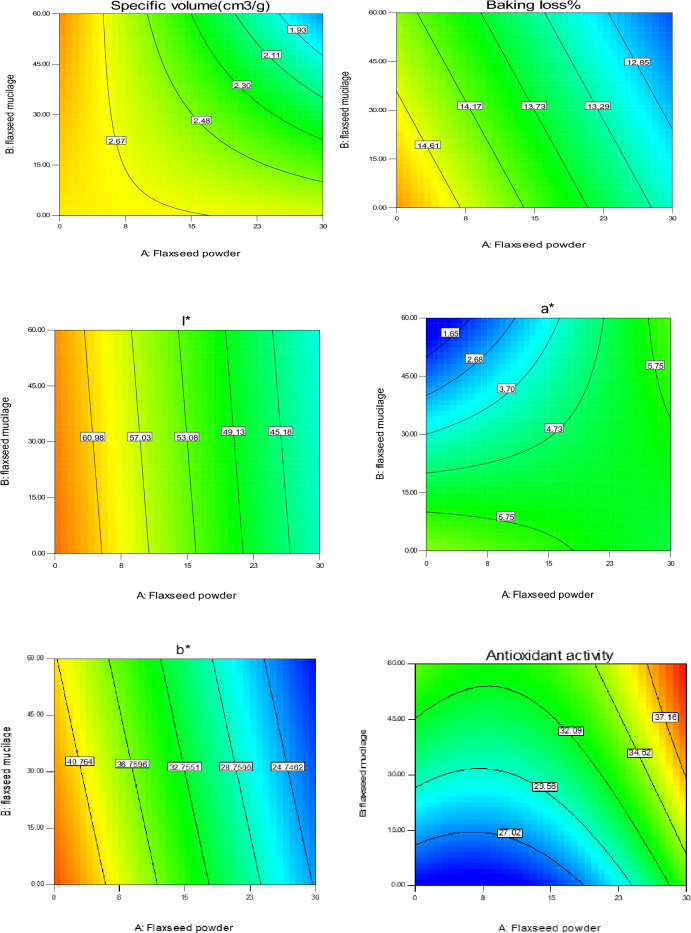
Contour plot of the effect of replacing flaxseed mucilage and flaxseed flour on baking loss, specific volume, antioxidant, and color parameters (l*, a*, b*) of sponge cake samples.

As depicted in Fig. 4, the antioxidant capacity of the samples increased with the replacement of FM and FF, and the highest amount of antioxidant capacity was observed in sponge cakes with the maximum incorporation of FM and FF. The increase in antioxidant capacity in samples containing flaxseed is due to the presence of antioxidant substances such as phenolic compounds and lignans. Flaxseed is a rich source of secoisolariciresinol diglucoside lignans and small amounts of matricinol lignans which can be converted into enterodiol and enterolactone lignans by colon bacteria^42^. Meral and Dogan (2013), confirmed the increase of antioxidant capacity in bread incorporated with flaxseed. Their study also found that the total phenols and flavonoids in bread increased significantly with the level of flaxseed^43^. Similar results about the increase of antioxidant capacity by the addition of flaxseed^43,44^ and eggplant flour^22^ in bread. The predicted model (Equation 17) for this response is a significant model with R^2^ and Adj-R^2^ of 0.98 and 0.96, respectively, and with a non-significant lack of fit (P > 0.05) showing the effectiveness of the model.

17 $$\text{Antioxidant \,capacity}=30.27+3.60\times A+3.75\times B-0.59\times AB+3.38\times {A}^{2}-0.73\times {B}^{2}$$

### Physical properties (weight loss, specific volume, and color)

As shown in Fig. 4, with the substitution of FM and FF, the weight loss percentage of the samples decreased (P < 0.05). It can be related to the ability of FM and FF to maintain the water because of their compounds mentioned, previously.

The specific volume of the sponge cakes decreased slightly with the replacement of FF and FM (Fig. 4). The reduction in volume with the increase of FF is due to the reduction of cake flour and as a result the reduction of gluten, which leads to the weakening of the flour and the reduction of the ability of the dough to retain air. The slight decrease in specific volume may be caused by the addition of mucilage, which may interrupt the protein network^19^. Also, the decrease in the specific volume of sponge cake with the increase in the substitution of FM can be attributed to the disruption of gas retention by fiber, the decrease in gas retention capacity, and the decrease in the amount of batter expansion during baking^19^. On the other hand, there is a possibility that the high levels of FM by producing more gel have strengthened the wall of the air bubbles entering the sponge cake batter so that these bubbles have lost the ability to expand and increase in volume during the baking process leading to a decrease in the specific volume of the samples^45^. Similar results about the reduction of specific volume with the replacement of different flour and mucilage have been reported in various research^14,19,21,23,45^. The regression model for specific volume (Equation 18) was significant (P < 0.05) with a non-significant lack of fit indicating the efficiency of the predicated model.

18 $$\text{Specific\, volume} =2.84-0.29\times A-0.18\times B-0.25\times AB$$

According to the results of the analysis of variance, the replacement of flaxseed and inulin had a significant effect on the color index of the samples (p ≤ 0.05). As can be seen in Fig. 4, the a*, b*, and L* values progressively decreased, increased, and decreased, respectively, by the incorporation of FF. Substitutional FM led to a decrease in a*-value and b* values in the sponge cake samples. The reason for the decrease in the L* index of the samples is due to the darker color of the FF (high mineral content) than the cake flour. The increase in the amount of a* value can be due to the presence of higher amounts of amino acids and free sugars in FF than in cake flour. Similar observations of color parameters were reported by FM and psyllium mucilage^4^ in cookies and FF^20^ in gluten-free biscuits.

### Texture properties and overall acceptance

**Figure 5:**
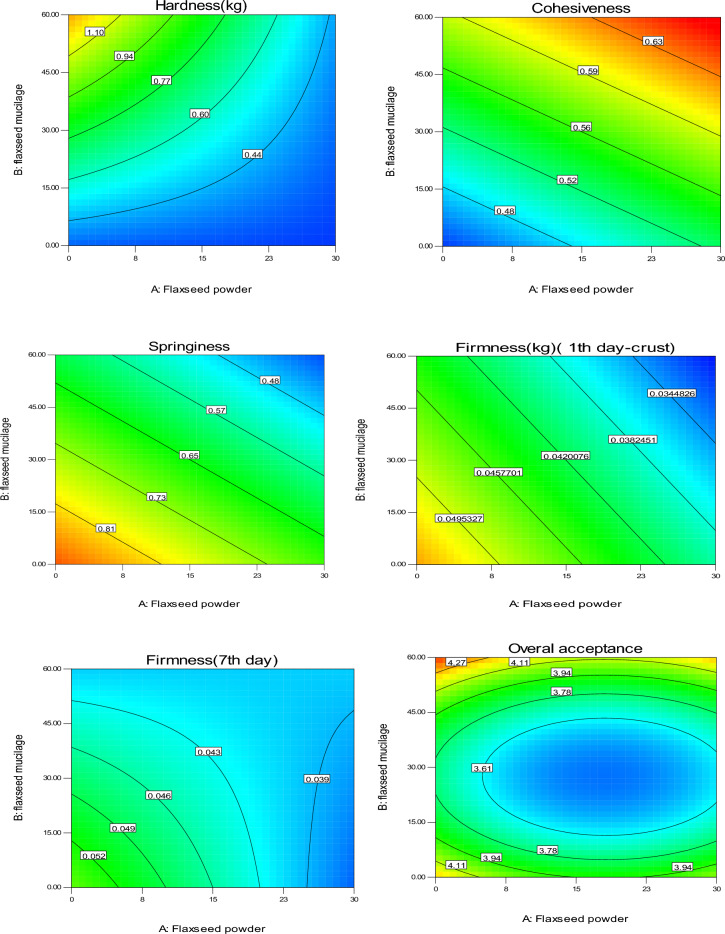
Contour plot of the effect of replacing flaxseed mucilage and flaxseed flour on the crumb hardness, cohesiveness, springiness, chewiness, crust firmness, and overall acceptance of sponge cake samples.

Figure 5 shows the contour plot of the effect of replacing FM and FF on crumb hardness, cohesiveness, springiness, chewiness, resilience, crust firmness, and overall acceptance of sponge cake samples. As a general trend, a higher substitution of FM led to a higher hardness. The increase in hardness was mainly due to a decrease in specific volume^21^, which aligns with the specific volume results. The increase in hardness at high levels of mucilage is also due to the increase in sponge cake dough viscosity because of the higher fiber content in the mucilage compared to animal oil. The incorporation of FF slightly decreased the crumb hardness. It may be related to the higher fat content of FF compared to the cake flour that had a plasticizer effect.

Cohesiveness as the internal resistance of the structure of the food determines the adhesion between the internal components^21^. The substitution of FM and FF increased sponge cake cohesiveness so that the highest amount was observed in samples with a higher substitution of FF and FM (Fig. 5). Similar results were reported by Avila et al. (2017) who stated that replacing the mixture of millet, quinoa, chickpea, flaxseed, and chickpea flours increased the cohesiveness of the sponge cake samples^24^.

Springiness determines elasticity by measuring the extent of recovery between the first and second compression^21^. The incorporation of sponge cake with FF and FM decreased springiness and crust firmness (1st and 7th day). This behavior is probably related to the larger size of FF compared to cake flour. In addition, FF contains high amounts of fiber, fat, and protein, which interact with gluten and starch affect the formation of the gluten network, and eventually cause a decrease in the springiness of the samples. A decrease in springiness was also reported with the incorporation of tea powder into sponge cake^25^ and chickpea fiber into bread^38^. The decrease in crust firmness may be related to the high moisture of the enriched samples as well as the gum state and the amount of fat in the flaxseeds. The results obtained were in agreement with the findings of Marpalle et al. (2014), who stated that the addition of FF to bread decreased the firmness of the samples^26^. Chewiness is a secondary parameter relying on the hardness. The chewiness is not affected by animal oil replacement with FM while it decreased with the incorporation of FF. This may be related to the lowering effect of FF on crumb hardness mentioned previously.

The predicted models for texture parameters (Equations 19–22) are significant models with a non-significant lack of fit (P < 0.05) showing the efficiency of the presented model.

19 $${\text{Hardness}}=0.57-0.22\times A+0.26\times B-0.19\times AB$$

20 $${\text{Cohesiveness}}=0.55+0.03\times A+0.07\times B$$

21 $${\text{Springiness}}=0.64-0.10\times A-0.14\times B$$

22 $${\text{Firmness}}( 1\text{th \,day}-{\text{crust}})=0.04-0.006\times A-0.004\times B$$

According to Fig. 5, it can be seen that FF and FM had a significant single and interaction effect on the total acceptance score of the sponge cake. The overall acceptance of the samples increased with the replacement of FM which was noticeable in low levels of FM. While the incorporation of sponge cakes with FF slightly decreased the overall acceptance score. The highest overall acceptance score (4.1 and 4.3) was related to sponge cakes with a high FM together with low FF, as well as samples with a low substitution of FM and FF, respectively. According to the results obtained from physical tests (specific volume, and colorimetric) and textural properties, this result is not far from expected. However, all studied samples had an acceptable overall acceptance score (> 3.6). Ganorkar and Jain (2014) cited the darkening of the color, the creation of a dry surface, the reduction of crispness, and the creation of a rougher mouthfeel as the reasons for the decrease in the overall acceptance score with the increase in the amount of FF^27^. The predicted model for this feature (Equation 23) is a significant model with R^2^ and Adj-R^2^ of 0.80 and 0.67, respectively, with a non-significant lack of fit (P > 0.05) showing the efficiency of the presented model.

23 $$\text{Overall \,acceptance}=3.46-0.08\times A+0.09\times B+0.004\times AB+0.21\times {A}^{2}+0.57\times {B}^{2}$$

### Optimizing the formulation

**Figure 6:**
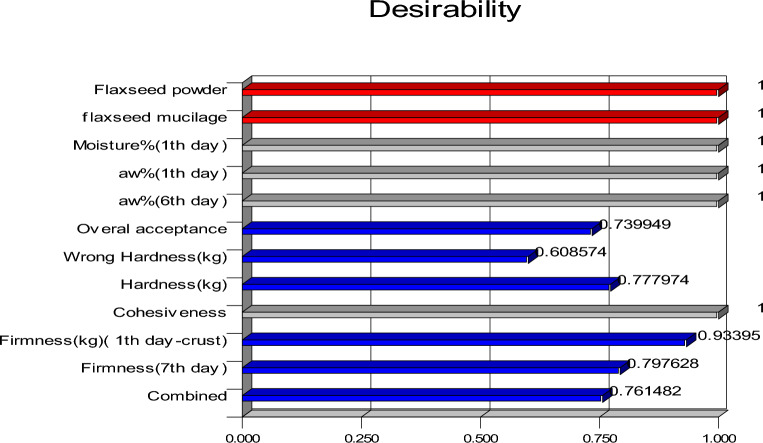
Optimization diagram and desirability function.

To determine the optimal processing conditions, the resulting models were numerically optimized for different responses. For this purpose, the desirability function is an effective method in multi-response optimization. The Optimum zone was found for 60% FM and 28% FF substitution in the sponge cakes (Fig. 6). In the following, some physicochemical characteristics were analyzed for the control and optimal sample, and their results were reported in Table 3. According to the results, the optimized sample had higher protein, ash, and moisture content compared to the control sample. While fat, peroxide, carbohydrate, and calorie of the optimized samples were lower than control samples. The reason for the reduction of fat could be due to the low amount of fat in FM (13.87%) compared to animal oil (80%) used in the formulation^4^. Other studies have also shown a decrease in fat with the addition of FF^28^ in composite flour and chia mucilage in cake and bread^19^. The high amount of protein and ash in FM (10.38%, 6.57%) and FF (27.46%, 3.96%), respectively leads to the production of sponge cakes with high nutritional value compared to the control^28^. The results were consistent with the results of Codina et al. (2019), who stated that the addition of flaxseed in wheat-flaxseed composite flour led to an increase in flour protein^46^. The addition of FF (4.69% moisture content) and mucilage (97.3% moisture content) increased the moisture content of the optimized sponge cake. This effect is due to the more minerals and fiber of FF compared to cake flour, so that fiber absorbs more water and increases the moisture content. Also, mucilage can form a continuous gel-like texture leading to an increase in the moisture content of the samples^47^. The reduction of peroxide can be explained by the reduction of fat in the formulation as a result of the substitution and the presence of hydroxyl groups in the mucilage, which have free radical trapping properties and thus prevent oxidation or reduce the rate of oxidation. The results obtained are in agreement with the results of Goyat et al. (2019)^48^. In general, in 60% FM + 28% FF sponge cake (optimal sample), protein increased by 15% and fat content, peroxide value, and calorie decreased by 22%, 26%, and 25%, respectively compared to the control one.

**Table 3:** Comparison of fat, protein, ash, moisture and peroxide of optimal and control sponge cake samples.

Factor	Protein	Fat	Ash	Moisture	PV (1st day)	PV (8th day)	PV (16th day)	Carbohydrate	Calorie(Kcal)
Control	6.85 ± 0.001^a^	22.87 ± 0.01^b^	0.55 ± 0.03^a^	22.1 ± 0.001^a^	1.72 ± 0.01^b^	1.84 ± 0.001^b^	2.10 ± 0.001^b^	47.78 ± 0.01^b^	424.15 ± 0.001^b^
28FF + 60FM	7.88 ± 0.002^b^	17.7 ± 0.03^a^	0.82 ± 0.03^b^	31.3 ± 0.001^b^	1.56 ± 0.01^a^	1.68 ± 0.001^a^	1.55 ± 0.001^a^	32.3 ± 0.01^a^	320.02 ± 0.001^a^

Same letters in a column indicate a significant difference (p ≤ 0.05).

*FM* Flaxseed mucilage *FF* Flaxseed flour.

## Conclusion

The present research is an example of the production of fortified low-calorie sponge cake containing FM and FF. In this research, sponge cakes were prepared in 13 treatments, and the physicochemical, sensory, and textural properties of the samples were measured. The results of rheological properties indicate Newtonian behavior in FM and pseudoplastic behavior in animal oil. The results of the sponge cake experiment showed that with the incorporation of the FM and FF, the moisture content, water activity, antioxidant capacity, and cohesiveness increased while the specific volume, springiness, and firmness of the crust decreased (P < 0.05). In this study, the optimization of fortified low-calorie cake formulation with the combination of 40% animal oil + 60% FM and 72% cake flour + 28% FF with a desirability of 0.76 and with favorable sensory, textural, and nutritional properties was carried out and confirmed. FM and FF in optimized sponge cake increased the nutritional value of the product. Therefore, FF and FM proved to be new alternatives for replacing cake flour and fat in food products, preserving the quality attributes and making them healthier foods.

## Data Availability

The datasets generated during and/or analyzed during the current study are not publicly available but are available from the corresponding author on reasonable request.
